# Successful application of World Health Organization multimodal strategy for hand hygiene

**DOI:** 10.1186/1753-6561-5-S6-P256

**Published:** 2011-06-29

**Authors:** JM Alsalman, Jameela Alsalman, Fatima Isa, Bahija Ahmed, Zaineb Alrayees, Khadmiya Altitoon, Ameena Al Sayegh, Najat Salem

**Affiliations:** 1 Infectious Disease / Medical, Ministry of Health, Manama, Bahrain

## Introduction / objectives

Hand hygiene is an effective means of preventing hospital-associated infection (HAI). Compliance among health care workers( HCWs) is the main strategic goal for the infection control committee as per the Ministry of Health collaboration with World Health Organization's (WHO) Global Patient Safety Challenge since October 2005.

## Methods

The following steps were taken by the infection control team in A secondary/tertiary care hospital (>1000 bed) and in a psychiatry hospital (>230 beds), for the last 3 years; were we:

- Acknowledged the importance of HAI and hand hygiene and started the surveillance.

- Developed ongoing campaigns at national or sub-national levels to promote and improve hand hygiene among HCWs; where we ran two campaigns per year

- Made information available on HAI and hand hygiene for all HCWs through educational sessions, materials, newsletter, workshops and a designed website.

- Shared experiences with the WHO

- Used the WHO strategies, guidelines and tools to tackle HAI

- Promoted the highest standards of practice and behavior to reduce HAI;

- Encouraged the senior management support for implementation of interventions to reduce HAIs.

- Created a voluntary team of medical residents to participate in the audit.

## Results

HCWs compliance with hand hygiene recommendations was low during the first Audit less than 10% (2007), with differences between doctors and nurses. Several activities introduced for HCWs during the whole year and on 5^th^ of each month. A group developed a hospital campaigns using the WHO multimodal strategy and observational tools. Opportunities for hand hygiene were audited during three years period. The compliance were reviewed which showed a significant increase in the compliance above 70% in 2010.

## Conclusion

The application of WHO multimodal strategy for hand hygiene helped in increasing the compliance of HCWs with hand hygiene, aiming to reach 100%.

## Disclosure of interest

None declared.

